# A Pediatric Strategy for the Next Phase of the SARS–CoV-2 Pandemic

**DOI:** 10.3389/fped.2020.582798

**Published:** 2020-10-09

**Authors:** Danilo Buonsenso, Piero Valentini, Umberto Moscato, Walter Ricciardi, Damian Roland

**Affiliations:** ^1^Department of Woman and Child Health and Public Health, Fondazione Policlinico Universitario A. Gemelli IRCCS, Rome, Italy; ^2^Dipartimento di Scienze Biotecnologiche di Base, Cliniche Intensivologiche e Perioperatorie, Università Cattolica del Sacro Cuore, Rome, Italy; ^3^Center for Global Health Research and Studies, Università Cattolica del Sacro Cuore, Rome, Italy; ^4^Istituto di Pediatria, Università Cattolica del Sacro Cuore, Rome, Italy; ^5^Sezione di Igiene, Istituto di Sanità Pubblica, Università Cattolica del Sacro Cuore, Rome, Italy; ^6^SAPPHIRE Group, Health Sciences, Leicester University, Leicester, United Kingdom; ^7^Paediatric Emergency Medicine Leicester Academic Group, Children's Emergency Department, Leicester Royal Infirmary, Leicester, United Kingdom

**Keywords:** COVID-19, SARS-CoV-2, children, policy, vaccination

## Abstract

Although the first wave of the SARS–CoV-2 pandemic relatively spared children, the next winter season will put a strain on health systems including pediatric services. Clinical staff managing children will need to deal not only with suspected cases of COVID-19, but also with the classic infectious agents that involve children during cold seasons. It will be necessary for physicians, institutions, policy makers, and families to prepare themselves for difficulties of this phase of the pandemic. Otherwise, the same problems experienced during the first wave of SARS–CoV-2, including shortages of human resources, personal protective equipment, and uncertainty, will be exacerbated by significant issues in hospital capacity. Here we highlight the potential role of improved vaccination services, school reorganization, home–outpatient–inpatients flows and telemedicine services in order to face the coming winter season.

## Introduction

**Figure 1:**
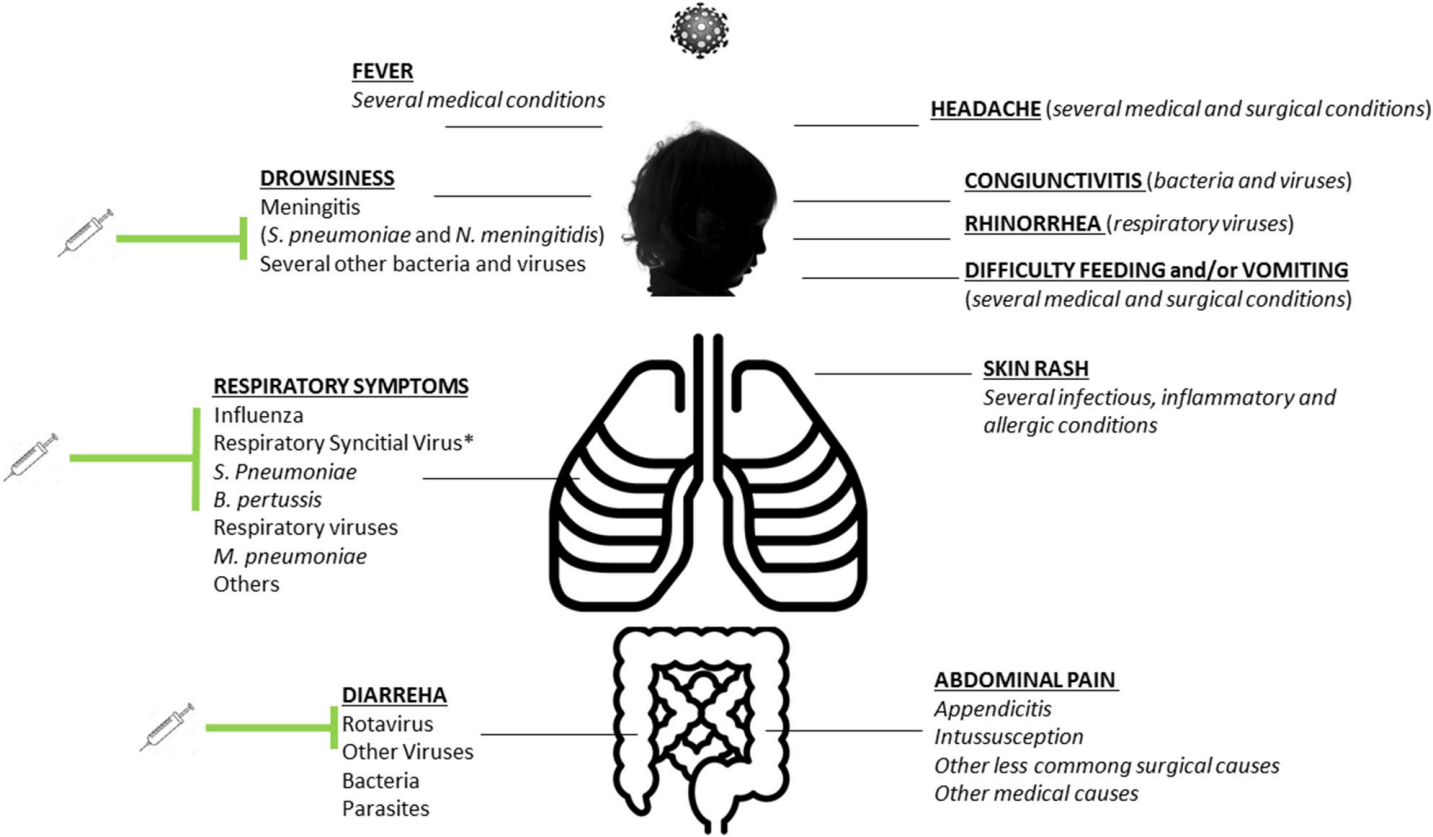
Summary of the most common signs and symptoms of SARS–CoV-2 infection in children and overlaps with other common infectious diseases in children. Preventable diseases with active immunization strategies (or with immunoglobulins in case of respiratory syncytial virus) are highlighted.

Initially described in China, SARS–CoV-2 rapidly spread all over the world being declared as a pandemic by the World Health Organization on March 11, 2020. Despite the virus causing millions of infections and thousands of deaths worldwide, the impact on children has been relatively mild. The number of infected children is much lower than in adults, and mortality is an extremely uncommon outcome in this age group (1). Although a more serious condition involving children has been recently described, the pediatric inflammatory multisystem syndrome temporally related with COVID-19 (PIMS-TS), this is a rare post–COVID-19 complication and in a minority of cases leads to death (2). As a likely result of parents' fear of going to hospital, the impact of lockdown, and reduced circulation of infectious diseases, the number of sick children presenting to hospital has dropped significantly, and most pediatric wards have been relatively empty (3). Although we do not know yet if there will be a second wave of COVID-19 in the next months, it is likely the pediatric practice will not be as unscathed as during the first wave of COVID-19. During the coming autumn and winter seasons in Europe, active surveillance will be necessary in order to understand if SARS–CoV-2 will circulate again and to prompt recognize new cases. This means that routine health care cannot be the same as in a pre–COVID-19 era. Importantly, the first COVID-19 wave began at the end of the winter seasons when influenza and bronchiolitis were diminishing and school closures strongly contributed to the reduction of other infectious diseases. In the next autumn, children will be readmitted at school, and the autumn–winter seasons will bring back the most common pathogens, such as influenza viruses, respiratory syncytial virus (RSV), pertussis, main bacterial pathogens (such as meningococcal and pneumococcal diseases, streptococcus, *Mycobacteria pneumoniae*), gastroenteritis, and other relatively less common but important pediatric pathogens. Considering that most of these conditions present several overlaps with SARS–CoV-2 (Figure 1), this will pose challenges to pediatricians and health system to appropriately manage all these conditions and properly allocate resources, because COVID-19 will need to be considered until exclusion, in order to reduce nosocomial transmission and new outbreaks.

Therefore, while the first wave relatively spared all services that see children, the next autumn/winter will be extremely difficult to be managed from a logistic and clinical point of view. Timely preparedness is needed to be ready to face this new scenario unlike for the initial pandemic (3). In particular, pediatricians and policymakers should focus on prevention of communicable diseases in children and reorganization of pediatric care, improving the logistic of inpatient and outpatients services, the appropriateness of access to outpatient and inpatient settings, the interaction between families and general practitioners and between GPs and hospitalists, and the role of telemedicine.

In this article, we suggest a number of preventive strategies that pediatricians, institutions, medical societies, and policy makers should address in order to appropriately face the near future, whether or not a second wave of SARS–CoV-2 will come.

## Policy Options and Implications, and Actionable Recommendations

### Vaccination

Among the possible preventive strategies aiming to reduce the rates of pediatric communicable diseases and indirectly reduce the overflow of children to outpatients/inpatients pediatric services, vaccinations would be the easiest, most effective, and fastest strategy available. Infants and young children are typically at high risk of admission to hospital after respiratory tract infection with viruses such as RSV and influenza virus, probably due to immaturity of the respiratory tract and immune system in this age group (4, 5). Therefore, an active vaccination strategy supported by communication strategies is now a public health priority.

The latest version of the recommended childhood and adolescent immunization schedules for 2020 has been released^1^. Although several countries have different policies, in most places the obligatory vaccinations are those against diphtheria, tetanus, pertussis, *Haemophilus influenzae* type b, and hepatitis B, whereas the others are recommended. However, this year will be of pivotal importance trying to achieve the highest immunization levels for all vaccinations, including measles, mumps, and rubella; meningococcal serogroups A, C, W, Y, and B; pneumococcal vaccination; and, extremely important, influenza. The highest rates should be achieved not only in the pediatric population, but also vaccination services should focus on all the family members (including pregnant women) and health care workers, in order to update catch-up immunizations and reach high levels of influenza immunizations among the health care workers. Currently, rates of influenza immunization are unacceptably low among health care providers. Typically, fewer than 70% of health care providers receive influenza vaccine^2^. Any of the mentioned preventable disorders has overlapping signs and symptoms with COVID-19; therefore, reducing the burden of these disorders will allow a more sustainable care within inpatient and outpatient services.

Sustainable systems for vaccinating children, adolescents, and adults must be developed in the context of a changing health care system in the era of COVID-19. Families should understand that now, other than the known advantages of vaccinations on child deaths, immunizations have the indirect effect of reducing the workload for a resource-limited health system. Most probably, a child with symptoms suggestive of an infectious disease will be considered possible COVID-19 cases for the next 6 months at least. This means that each child will be isolated, that only one caregiver will be allowed to be with the child during the clinical evaluation or admission, and that bed capacity will not allow the isolation of all sick children until COVID-19 is excluded with a high probability. A similar scenario will impair appropriate flows in the PEDs and admission wards, lengthen waiting times in the emergency departments, and ultimately impair or delay the appropriate care for children.

Importantly, high immunization rates cannot rest upon one-time or short-term efforts^2^. Parents and patients need educating about each recommended vaccine, the disease it prevents, and the indirect effects on the health system. This step to achieve the highest immunization rates is now more necessary than ever. Vaccine education and usefulness should be highlighted during each visit, *via* social media and official national communication channels, allowing parents to provide questions, receive answers, and discuss their concerns^3^. These strategies are neither novel or innovative communication strategies and therefore would require insignificant funds compared to the benefits to health system impact.

### School Reorganization

Multiple studies have shown serious consequences of COVID-19 in children are relatively rare, and there is growing evidence that children themselves are more resistant to carriage and transmission compared with adults (6). Somekh et al. (7) also confirmed these data examining the dynamics of COVID-19 transmission within families. They demonstrated significantly lower rates of COVID-19 positivity in children compared with adults residing in the same household. Children 5–17 years of age were 61% and children 0–4 years of age were 47% less likely to have positive microbiological results (7).

Although these results do not necessarily indicate that school reopening is safe, in any case children will need to be readmitted at school. Instruction is a priority right of every child and a fundamental instrument for the growth of every society; therefore, children cannot continue a home-only education process, because this can be associated with reduction in socialization skills, poorer education outcomes, reduced activity, inappropriate nutrition, visual problems, increase in domestic accidents (8), or violence.

Even assuming that children will need to be admitted back to school, there are no doubts that the school closure during this pandemic had an indirect benefit on the reduction of communicable diseases. French authors found a significant decrease (70%) of acute gastroenteritis, common cold, and acute otitis media compared to the expected values, and a 63.5% decrease in bronchiolitis. In general, a dramatic decrease in overall PEDs visits (−68.5%) and hospitalization (−44.7%) was observed (9). However, the reduction of PED presentation can also be due to different parents' decision-making motives and reasoning on bringing their child to hospital or not. During these months of the COVID-19 pandemic, an intense debate has started about possible late, belated, or “not-at-all” presentations in the PED related to the pandemic; however, strong evidences suggesting why the PED attendances have changed that much during the pandemic are still lacking (10).

Although the assumption that children have higher risks of contagion within close communities, such as schools, is well known among pediatricians, this is the first time in the recent history that this finding has been methodologically demonstrated. Although school closure aiming to reduce pediatric infectious disease burden is neither ethically feasible nor sustainable for families, the results from Angoulvant et al. (9) and Bressan et al. (3) provide unique evidence to implement new guidelines and new routines to provide child education at school. Currently, most classrooms are overcrowded with 20 or more students per class, and lunch is usually served in large common spaces where students from all the schools are collected to eat.

In light of new evidences and the need to reduce as much as possible the diffusion of infectious diseases among children during the next season (because this would lead to include all cases in the differential diagnosis with COVID-19 because of similar symptoms), a reorganization of school environments should be a priority for policy makers. Institution should plan to reduce the number of students in each room and practicing physical distance within the class, separating desks of 1 m from each other, and providing sanitizers at the entrance of each class. The reduction of students per class would also allow a better students-to-teacher ratio and potentially improve school outcomes. Also, the figure of a school medical doctor/nurse might be considered (or implemented if already in place) in order to detect early cases of suspected infectious diseases to be sent home and to ensure catch-up immunizations are up-to-date. Preparing schools will be a challenge both logistically and financially, and it is likely underlying inequities will be exacerbated with the most deprived settings likely to be disproportionately affected. However, given the importance of returning children to school, it may well be that governments prioritize education in a manner that they have not done so before.

### Child Health Care in the Early Post–COVID-19 Pandemic

**Figure 2:**
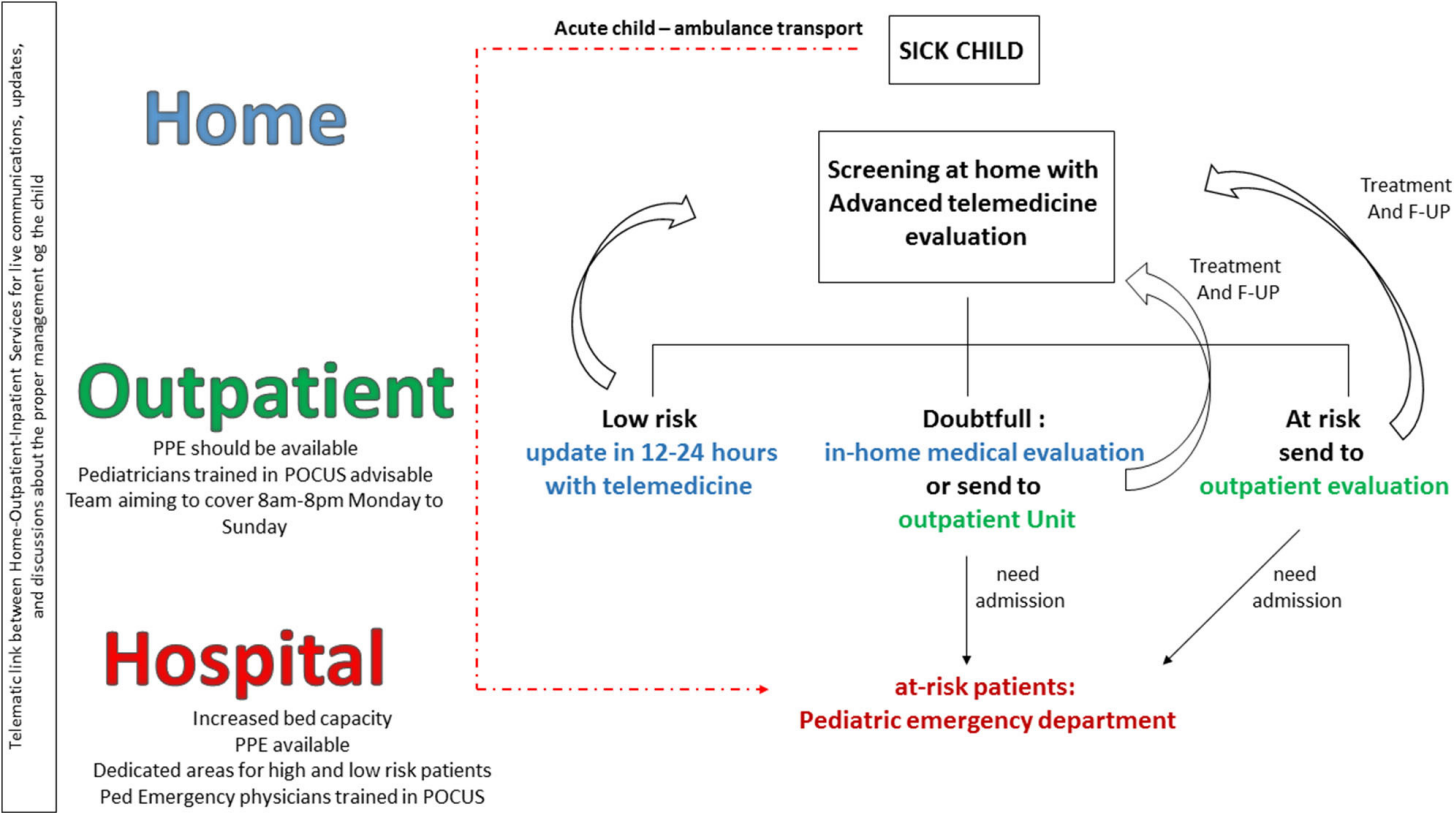
Child health care reorganization in the early post–COVID-19 pandemic. A better flow and interaction between families, family pediatrician/general practitioners, and hospital pediatricians, as well implementation of telemedicine services, are needed to prevent overflow of patients in the emergency departments and saturation of hospital resource.

The SARS–CoV-2 pandemic has significantly altered health care systems with shortages of personal protective equipment (PPEs) (3), human resources, and bed capacity not only related to intensive care unit ones. Emergency departments and admission wards changed their assets, allowing dedicated flows for high-risk and low-risk COVID-19 patients (11). Outpatient services have been postponed to the end of the peak unless necessary, and telemedicine services implemented in order to allow routine controls. In most countries, the peak of the SARS–CoV-2 arrived when the flu season was over or close to the end, and school closures determined a strong reduction of common pediatric infectious diseases. The reduction of common seasonal infections helped health systems focus most of their efforts on COVID-19. However, the next autumn/winter will probably not be the same, no matter whether a second wave of SARS–CoV-2 infection begins or not. Outpatient services cannot be postponed again, because a clear impact on other major contributors to mortality and morbidity, such as reduced number of new diagnoses of cancers and missed diagnosis, have been documented (12). Children will be back at school, and as discussed, the reoccurrence of common infections is expected. Therefore, even though the direct clinical impact of the SARS–COV-2 virus on children has been limited with a very low mortality rate, and the COVID-19–related pediatric inflammatory multisystem syndrome remains a relatively rare consequence of the disease, pediatricians will still need to include SARS–CoV-2 in the differential diagnosis. This will be necessary to assess the reoccurrence of SARS–CoV-2 and to prevent the spread of the virus in the health facilities and communities. Because parents/caregivers always accompany the child, they may need to be screened as well if the child needs hospital admission. In this context, a review of the necessity of regular outpatient follow-up, family education about the management of fever in children and recognition of red flags, implementation of technology, and more appropriateness in the access to emergency departments is needed (Figure 2) (13).

Ideally, parents of every sick child should be able to have relatively prompt access to their family pediatrician (FP) or general practitioner (GP) before bringing the child to medical attention, unless critical conditions that require immediate access to the hospital by ambulance (Figure 2). Pediatricians should develop screening and telematics visit forms to perform a first evaluation of a child with telemedicine. This televisit should allow the pediatrician to discuss with both the mother and child, see how the child behave, and take an appropriate clinical history. At this moment, the FP/GP should decide if the child can be reassessed with telemedicine in 12–24 h or a real visit should be performed, at home or in the outpatient service. Depending on the health system model of that country, the outpatient service and up-to-date FP/GP should be able to perform a comprehensive examination. This may include the use of point of care ultrasound, assessing major causes of diseases including pneumonia, appendicitis, intussusception, and pyloric stenosis, which can be easily diagnosed at point of care by trained physicians or other near patients testing interventions. Only after this examination, the child should be sent to hospital if needed. This would be a radical change of service flow in some settings and may require significant short-term investment in training and capacity. However, to not do this may confront services with an impossible capacity challenge. Real-time platforms should allow the communication between the FP/GP and hospital pediatrician in order to share clinical decisions and agree with the most appropriate flow of the single patient. This last point will be necessary also for the follow-up of children with documented SARS–CoV-2 infection or known exposure that does not initially deserve admission because of their clinical condition. We now know that a minority of these children will develop PIMS-TS weeks after the initial exposure to the virus; therefore, family education, telemedicine daily updates, and proper interaction between family, FP/GP, and hospital will be of pivotal importance.

## Conclusions

The next winter season will put a strain on the health system, including all child health services. It is necessary that physicians, institutions, policy makers, and families all together prepare themselves on time to face in the best way the difficulties of the near future; otherwise, we will find ourselves facing the same problems experienced during the first wave of SARS–CoV-2, including shortages of human resources, PPEs, and hospital capacity. In this context, improved vaccination services, school reorganization, a renewed concept of health system with appropriateness of the home–outpatient–inpatients flows, and strengthening of telemedicine services are public health priorities, right now.

## Author Contributions

DB and DR has the initial concept and wrote a preliminary draft. PV contributed to the writing of the clinical overview of the COVID-19 in children. UM actively work in immunizations and contributed in the writing of the immunization strategies. WR provided his expertise in the proposal of new future strategies to be prepared in facing the next phase of SAR-CoV-2 pandemic in children. The collaboration between experts in different fields from different countries was pivotal in writing a comprehensive manuscript on a particularly important topic on child health. All authors contributed to the article and approved the submitted version.

## Conflict of Interest

The authors declare that the research was conducted in the absence of any commercial or financial relationships that could be construed as a potential conflict of interest.

## Footnotes

^1^Available online at: https://redbook.solutions.aap.org/SS/Immunization_Schedules.aspx (accessed June 18, 2020).

^2^Available online at: https://www.cdc.gov/vaccines/pubs/pinkbook/strat.html (accessed June 18, 2020).

^3^Available online at: https://www.aap.org/en-us/advocacy-and-policy/aap-health-initiatives/immunizations/Practice-Management/Pages/office-strategies.aspx (accessed on June 18, 2020).
